# Expression and significance of lncRNAs derived from PBMC in rheumatoid arthritis

**DOI:** 10.3389/fimmu.2025.1515665

**Published:** 2025-03-20

**Authors:** Xiaoke Yang, Zhongling Yang, Ziqiang Shuai, Mingming Zhang, Sheng-qian Xu, Zong Wen Shuai

**Affiliations:** ^1^ Department of Rheumatology and Immunology, the First Affiliated Hospital of Anhui Medical University, Hefei, Anhui, China; ^2^ Department of Sports Injury and Arthroscopic Surgery, the First Affiliated Hospital of Anhui Medical University, Hefei, Anhui, China

**Keywords:** rheumatoid arthritis, PBMC, long non-coding RNA, biomarker, pathogenesis

## Abstract

**Background:**

Long non-coding RNAs (lncRNAs) are gaining recognition for their critical involvement in diverse autoimmune disorders. Nevertheless, reseach investigating the role of lncRNAs in rheumatoid arthritis (RA) is relatively scarce.

**Methods:**

Comprehensive transcriptome sequencing was executed to acquire a lncRNA expression pattern in peripheral blood mononuclear cells (PBMC) of RA. Then, we confirmed the sequencing data by real-time quantitative polymerase chain reaction (RT-qPCR).

**Results:**

The findings showed decreased levels of LINC00494, TSP0AP1-AS1, MCM3AP-AS1 and LINC01588, increased levels of OIP5-AS1, in PBMC of RA compared to controls. ROC analysis for the five dysregulated lncRNAs demonstrated an area under curve (AUC) extending from 0.654 to 0.915, and their combination had high utility for accurate RA diagnosis (AUC = 0.920). There existed a negative relation between RF and LINC00494 expression (*P*=0.027), positive relation between anti-CCP and MCM3AP-AS1 (*P*=0.024), and negative relation between CRP and LINC01588 expression (*P*=0.020).

**Conclusions:**

Our study indicated that LINC00494, TSP0AP1-AS1, MCM3AP-AS1, LINC01588 and OIP5-AS1 in PBMC may be the biomarkers for RA.

## 1 Introduction

Rheumatoid arthritis (RA) is a chronic multisystem autoimmune disorder with the features of synovitis, symmetrical pain of joints, cartilage and bone destruction as well as impaired mobility, potentially resulting in long-lasting disability and severely compromising patients’ overall wellbeing (1, 2). RA affects about 1% of the global population, with the incidence possibly higher among Europeans and Asians (3). At present, the exact mechanisms underlying RA pathogenesis remain unclear. It is now recognized that a complex interplay between genetic predisposition, epigenetic influences, environmental exposures, and immune system dysregulation contributes to the development of RA (4). Although genome-wide analyses have identified multiple susceptibility loci associated with RA, genetic factors can only explain about 15% of the etiology, and epigenetic inheritance, transcriptional and post-transcriptional regulation also play an important role in RA. Particularly, Genome-wide analyses have identified numerous genetic loci conferring susceptibility to RA. Notably, while approximately 10% of transcribed RNA molecules code for proteins, the remaining 90% constitute non-coding RNAs (ncRNAs) that exhibit minimal or devoid of the ability to encode proteins (5).

Long non-coding RNAs (lncRNAs) as crucial components of epigenetic mechanism, are a subset of ncRNAs surpassing 200 nucleotide units in dimension, performing indispensable functions across diverse biological processes (6, 7). These transcripts contribute to joint homeostasis through their involvement in epigenetic modifications, regulating gene transcription, and influencing post-transcriptional processes. Thus, dysregulation of lncRNAs can trigger joint inflammation and worsen joint damage (8). Recently, increasing evidence have indicated that lncRNAs act as a critical role in the development of autoimmune diseases like RA.

The lncRNA NEAT1 was found to be increased in RA peripheral blood mononuclear cells (PBMC) and Th17 cell while promoting Th17 cell differentiation by reducing ubiquitylation of STAT3 (9). Besides, NEAT1 was overexpressed in RA PBMC derived exosomes and stimulated the hyperplasia of fibroblast-like synoviocytes (FLS) while driving inflammatory processes via regulating the miR-23a/MDM2-SIRT6 pathway (10). LncRNA H19, upregulated in RA-FLS and synovium, was found to promote inflammation and joint destruction by sponging miR-103a which decrease the expression of IL15 and DKK1 (11). Additionally, the lncRNA PINT, which is downregulated in RA-FLS, moreover, it can facilitate the cell multiplication, invasion, and the release of cytokines associated with inflammation by blocking the ERK pathway (12). However, the abundance of these lncRNAs in patients with RA are still a subject of debate, and their underlying pathogenic mechanisms have not yet been fully clarified. Hence, to research the expression patterns and functional mechanisms of RA has great novelty.

The current investigation adopted an innovative approach, employing comprehensive transcriptome analysis to characterize lncRNA expression patterns in PBMC from RA patients. Subsequently, we utilized real-time quantitative polymerase chain reaction (RT-qPCR) to validate differentially expressed lncRNAs in PBMC samples. Further, we evaluated their potential utility as RA diagnostic markers and explored correlations with RA disease indicators.

## 2 Methods

### 2.1 Participants

The study employed a two-phase case-control design. The initial phase included three new-onset RA patients and three sex-age matched healthy controls (HCs) for lncRNAs screening. Then, 38 RA patients and 36 sex-age matched HCs were subjected to detect the lncRNAs expression in the validation phase. All enrolled cases having RA were selected from the First Affiliated Hospital of Anhui Medical University and had to fit the 2010 ACR/EULAR criteria (13). The healthy people were checked to make sure they didn’t have a record of RA or other self-immune maladies. We collected the necessary information from questionnaires and medical records. Informed consent followed the Helsinki Declaration was acquired from all participants. This research got permission from the Ethics Committee of the First Affiliated Hospital of Anhui Medical University (2022316).

### 2.2 RNA extraction

For this study, 10 ml of blood was taken from each person. Then, PBMC were separated from the blood using a method called Ficoll density gradient centrifugation and kept at -80°C. After that, all the RNA was taken out of the PBMC by trizol reagent (Invitrogen, CA, USA). The scientists followed the steps given by the company to make sure they did it correctly. Then, they used NanoDrop One spectrophotometer (Thermo Scientific in the USA) to check RNA quantity and purity.

### 2.3 Comprehensive transcriptome sequencing

LC-Bio Technology Co., Ltd. (Hangzhou, China) conducted the comprehensive transcriptome sequencing on the Illumina Hiseq 2500 platform. The threshold *P* < 0.05 plus log2 FC> 1.0 was used as the selection criteria to identify the up-regulated and down-regulated lncRNAs. Cluster analysis was utilized to identify variations in lncRNA expression patterns across the examined samples.

### 2.4 LncRNA verification by real-time quantitative polymerase chain reaction

RNA was reverse transcribed into cDNA using the PrimeScript™RT reagent Kit manufactured by Takara, Japan. We got primers for seven candidate lncRNAs (LINC00494, TSP0AP1-AS1, MCM3AP-AS1, LINC01588, OIP5-AS1, THUMPD3-AS1 and LINC01094) from Sangon Biotech in Shanghai. In addition, RT-qPCR was conducted to detect the levels of 7candidate lncRNAs in PBMC by SYBR Green (Takara, Japan). The results were described as relative levels of target lncRNAs standardized to housekeeping gene β-actin and were determined utilizing the 2^-ΔΔCt^ approach.

### 2.5 Statistical analysis

SPSS 26.0 (from a company called IBM in NY, USA) and GraphPad Prism 9.5 (GraphPad Software, San Diego, CA, USA) constituted the statistical examination’s foundation. Continuous variables were articulated as mean ± SD, and categorical variables as median (interquartile range). When the numbers were normal, we used the t-test to compare if there was a difference between two groups. When the numbers were non-normal, we used the Mann-Whitney U test. In this study, the ROC curve analysis, along with the calculation of the area under the curve (AUC), was conducted to assess the potential diagnostic utility of the lncRNAs. Spearman rank test was applied to analyze the association in lncRNAs and RA symptoms. The critical value of statistical significance was *P*-value that was below 0.05.

## 3 Results

### 3.1 Features of the study objects

In our study, an aggregate of 41 RA cases and 39 sex-age matched HCs were divided into two phases. Three RA cases and three HCs were enrolled in the screening sequencing phase, while 38 RA cases and 36 HCs were subjected in the preliminary verification stage. The relevant characteristics were exhibited in **Table 1**.

**Table 1:** Demographic and clinical characteristics of RA patients and healthy controls.

Phase	Demographic and clinical characteristics	RA	HC	*P*
Screening				
	n	3	3	
	Age (years, mean±SD)	43.35±5.13	45.67±7.88	0.513
	Gender (male/female)	0/3	0/3	1.000
	Disease duration (years, mean±SD)	3.49±1.40	/	
	ESR (mm/h, mean±SD)	50.00±20.82	/	
	CRP (mg/L, mean±SD)	50.98±31.46	/	
	RF (IU/ml, mean±SD)	91.23±51.84	/	
	Anti-CCP(U/ml, mean±SD)	302.33±283.42	/	
	DAS28(mean±SD)	4.41±0.39	/	
Validation				
	n	38	36	
	Age [years, M (P25, P75)]	49.27(43.78,65.68)	52.61±1.69	0.880
	Gender (male/female)	4/34	6/30	0.443
	Disease duration [years, M(P25, P75)]	1.54(0.29,7.62)	/	
	ESR [mm/h, M (P25, P75)]	37.00(19.50,59.25)	/	
	CRP[mg/L, M (P25, P75)]	18.29(10.98,61.28)	/	
	RF [IU/ml, M (P25, P75)]	83.70(39.85,268.53)	/	
	Anti-CCP[U/ml, M (P25, P75)]	202.65(59.05,706.50)	/	
	DAS28(mean±SD)	4.99±1.21	/	

Anti-CCP, anticyclic citrullinated peptide; CRP, C-reactive protein; DAS28, disease activity score of 28 joints; ESR, erythrocyte sedimentation rate; HC, healthy control; RA, rheumatoid arthritis; RF, rheumatoid factor; SD, standard deviation.

### 3.2 LncRNA and mRNA expression patterns in RA

**Figure 1:**
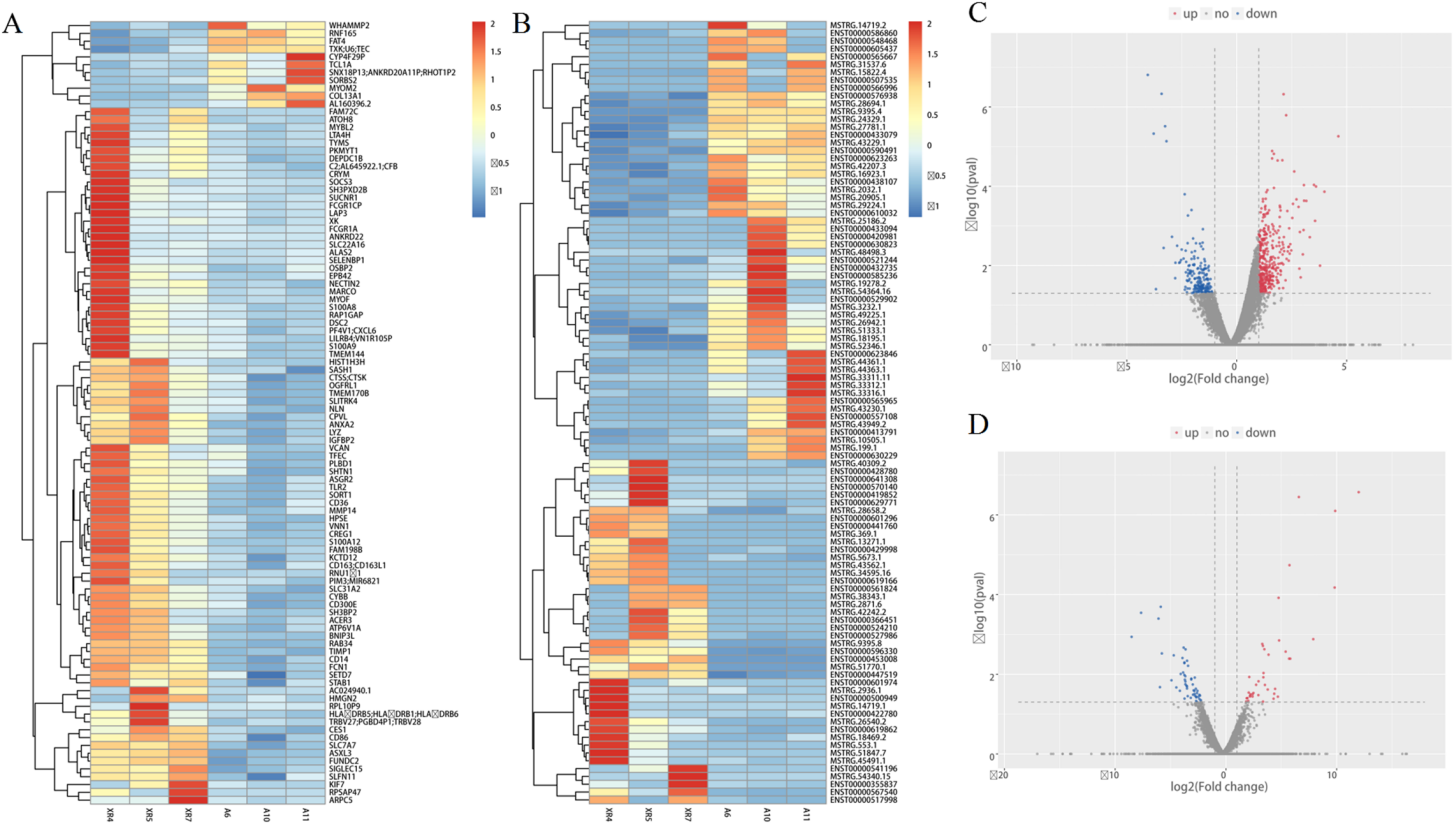
Comprehensive transcriptome sequencing analysis in lncRNAs and mRNAs expression between RA patients and HCs. **(A, B)** heat map depicting the differentially expressed lncRNAs and mRNAs; **(C, D)** volcano plot of differentially expressed lncRNAs and mRNAs. Red indicates high expression, blue indicates low expression, and grey indicates non significant.

Comprehensive transcriptome sequencing result indicated that 114 lncRNAs and 561 mRNAs were different between RA cases and HCs. The former includes 64 upregulated lncRNAs and 50 downregulated lncRNAs, whereas the latter includes 373 upregulated mRNAs and 188 downregulated mRNAs (**Figure 1**).

**Figure 2:**
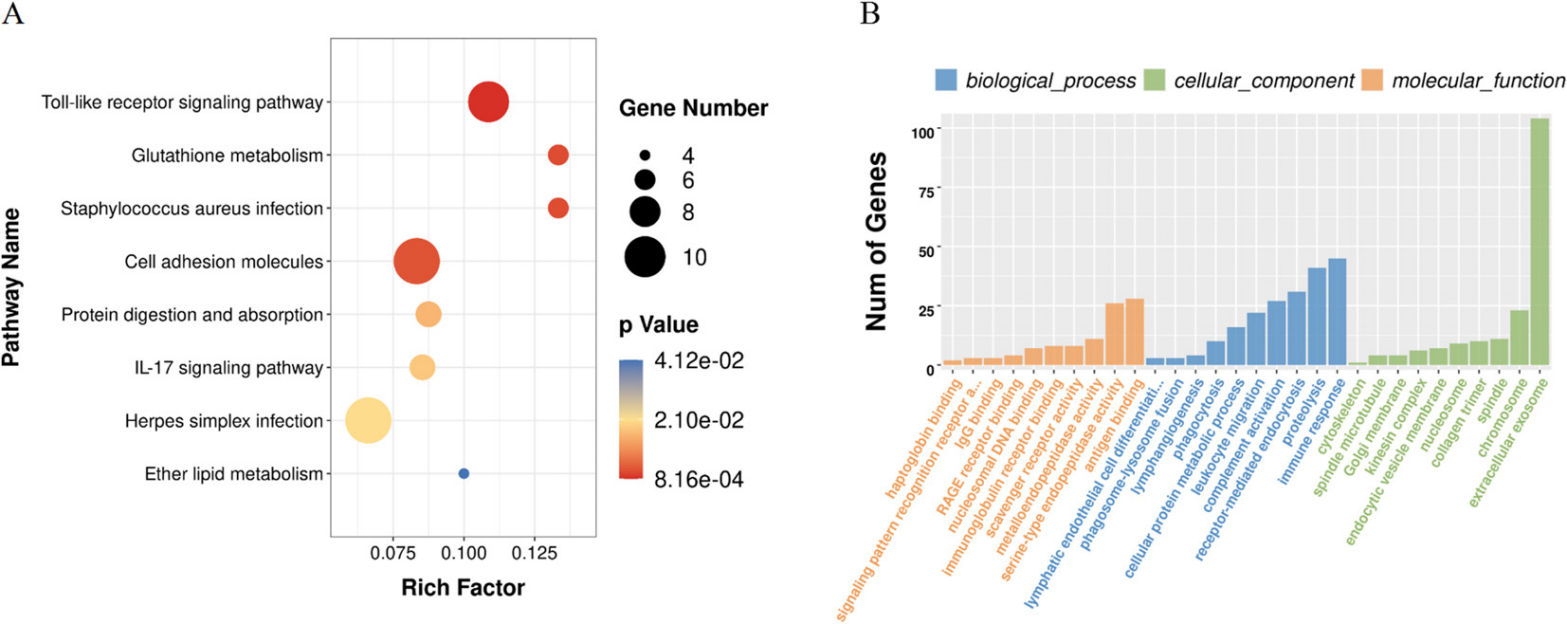
Enrichment analysis of the differentially expressed mRNAs: **(A)** KEGG pathway analysis; **(B)** functional annotation based on Gene Ontology.

KEGG analysis showed the top three pathway were toll-like receptor signaling, glutathione metabolism, and staphylococcus aureus infection. In GO analysis, the three most prominent molecular function encompassed antigen binding, serine-type endopeptidase activity, and immunoglobulin receptor binding. The top three biological processes terms were complement activation, receptor-mediated endocytosis, and immune response. Lastly, the three major cellular constituents were mostly in extracellular exosome, collagen trimer, and nucleosome (**Figure 2**).

### 3.3 Validation by RT-qPCR

**Figure 3:**
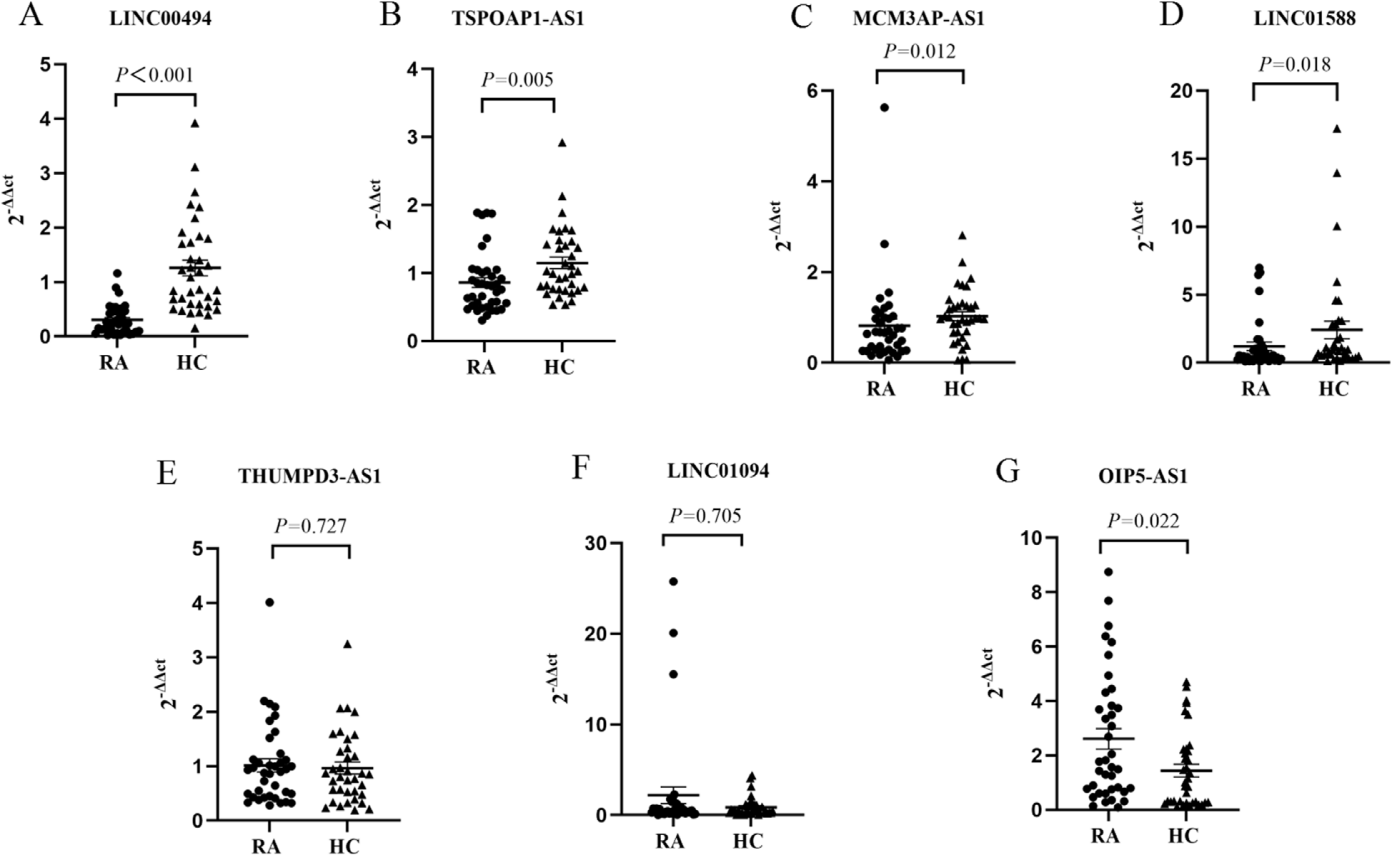
The expression levels of **(A)** LINC00494, **(B)** TSP0AP1-AS1, (C) MCM3AP-AS1, **(D)** LINC01588, **(E)** THUMPD3-AS1, **(F)** LINC01094, and **(G)** OIP5-AS1. HC, healthy control; RA, rheumatoid arthritis.

Using the threshold *P*< 0.05 plus log2 FC> 1.0 as the selection criteria, seven candidate lncRNAs (LINC00494, TSP0AP1-AS1, MCM3AP-AS1, LINC01588, OIP5-AS1 and THUMPD3-AS1, LINC01094) were selected for further verification by RT-qPCR in the PBMC of 38 cases with RA and 36 HCs (**Table 2**). Among them, LINC00494, TSPOAP1-AS1, MCM3AP-AS1 and LINC01588 in RA were found down-regulated, and OIP5-AS1 was up-regulated in RA. However, our findings indicated that the expression levels of THUMPD3-AS1 and LINC01094 had no obvious difference between RA subjects and HCs (**Table 3**) (**Figure 3**).

**Table 2:** The expression of 7 candidate lncRNAs between RA (n = 3) and HC (n = 3) in the screening phase.

lncRNA	Log2 fold change (RA vs. HC)	*P* (RA vs. HC)
LINC00494	-4.915214058	0.014*
TSPOAP1-AS1	-4.154844348	0.026*
MCM3AP-AS1	-3.746425857	0.005*
LINC01588	-2.779171230	0.035*
THUMPD3-AS1	7.887341225	0.001*
LINC01094	4.302550571	0.041*
OIP5-AS1	3.471869771	0.002*

HC, healthy control; RA, rheumatoid arthritis. *: the result of the Mann-Whitney U test between RA and HC groups.

**Table 3:** The expression of 7 candidate lncRNAs between RA (n = 38) and HC (n = 36) in the validation phase .

lncRNA	RA	HC	*P*
LINC00494	0.237(0.100,0.472)	0.978(0.595,1.778)	<0.001*
TSPOAP1-AS1	0.783(0.515,1.036)	1.009(0.765,1.457)	0.005*
MCM3AP-AS1	0.650(0.268,1.004)	0.976(0.671,1.269)	0.012*
LINC01588	0.452(0.249,1.077)	0.907(0.410,2.857)	0.018*
THUMPD3-AS1	0.914(0.446,1.150)	0.829(0.493,1.316)	0.721
LINC01094	0.449(0.265,1.305)	0.424(0.261,1.099)	0.697
OIP5-AS1	1.666(0.732,3.952)	0.945(0.266,2.208)	0.022*

HC, healthy control; RA, rheumatoid arthritis. *: the result of the Mann-Whitney U test between RA and HC group.

### 3.4 Prospective diagnostic utility of five distinctly expressed lncRNAs in RA

**Figure 4:**
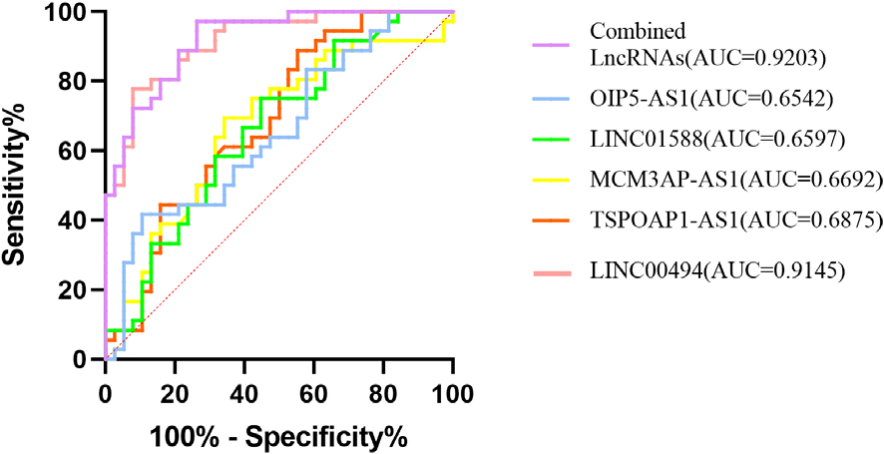
ROC curves of the five differentially lncRNAs for their AUC calculations.

ROC analysis was applied to explore the clinical value of five differentially expressed lncRNAs (LINC00494, TSPOAP1-AS1, MCM3AP-AS1, LINC01588 and OIP5-AS1) in the diagnosis of RA. As shown in **Table 4**; **Figure 4**, the AUC of the five lncRNAs in RA patients were ranged from 0.654(0.529,0.779) for OIP5-AS1 to 0.915(0.853,0.976) for LINC00494. The sensitivity in RA varied between 41.67% (OIP5-AS1) and 88.89% (TSPOAP1-AS1), and the specificity varied between 44.74% (TSPOAP1-AS1) and 92.11% (LINC00494). The AUC of the five differentially expressed lncRNAs combined was 0.920(0.862,0.979), the sensitivity was 97.22%, and the specificity was 73.68%.

**Table 4:** Potential diagnostic value of the five differentially expressed lncRNAs for RA.

lncRNA	AUC	95%CI	*P*	Sensitivity(%)	Specificity(%)
LINC00494	0.915	(0.853,0.976)	<0.001*	77.78%	92.11%
TSPOAP1-AS1	0.688	(0.567,0.809)	0.005*	88.89%	44.74%
MCM3AP-AS1	0.669	(0.544,0.795)	0.012*	69.44%	65.79%
LINC01588	0.659	(0.536,0.784)	0.018*	75.00%	55.26%
OIP5-AS1	0.654	(0.529,0.779)	0.022*	41.67%	89.47%
Combined lncRNAs	0.920	(0.862,0.979)	<0.001*	97.22%	73.68%

AUC, area under the curve; CI, confidence interval; RA, rheumatoid arthritis; ROC, receiver operating characteristic curve.

### 3.5 Correlation between the five dysregulated lncRNAs and RA-related clinical indices

The correlation analysis of the five dysregulated lncRNAs with the laboratory indicators of RA patients were summarized in **Table 5**. Rheumatoid factor (RF) levels demonstrated a negative correlation with LINC00494 expression (rs=-0.359, *P*=0.027). Meanwhile, the level of anti-cyclic citrullinated peptide (anti-CCP) antibodies exhibited a positive correlation with MCM3AP-AS1 expression (rs=0.366, *P*=0.024), and C-reactive protein (CRP) levels exhibited an inverse relationship with LINC01588 expression (rs=-0.375, *P*=0.020). Whereas, we didn’t find any connection between these lncRNAs and two other parameters, erythrocyte sedimentation rate (ESR) and disease activity score of 28 joints (DAS28).

**Table 5:** Relationship between the five differentially expressed lncRNAs and RA-related clinical indices.

Clinical index	LINC00494		TSPOAP1-AS1		MCM3AP-AS1		LINC01588		OIP5-AS1	
	rs	*P*	rs	*P*	rs	*P*	rs	*P*	rs	*P*
ESR	-0.077	0.644	-0.043	0.799	-0.065	0.699	-0.202	0.223	0.044	0.794
CRP	-0.166	0.320	-0.175	0.294	-0.042	0.804	-0.375	0.020*	0.209	0.208
RF	-0.359	0.027*	-0.206	0.214	-0.114	0.497	-0.223	0.179	0.092	0.581
Anti-CCP	0.035	0.833	0.197	0.236	0.366	0.024*	0.278	0.091	0.256	0.120
DAS28	0.114	0.494	0.055	0.743	0.102	0.541	0.086	0.608	0.067	0.690

Anti-CCP, antibody against cyclic citrulline polypeptide; CRP, C reactive protein; DAS28, disease activity score of 28 joints; ESR, erythrocyte sedimentation rate; RF, rheumatoid factor. *: *P* < 0.05.

## 4 Discussion

Accumulating evidence highlights the distinct roles of lncRNAs in modulating immune cell activation and differentiation, with implications in autoimmune disease pathogenesis (14). Our study’s high-throughput RNA sequencing results demonstrated the dysregulation of 114 lncRNAs in PBMC from RA patients, with 64 upregulated and 50 downregulated. Subsequent RT-qPCR validation identified seven candidate lncRNAs, of which LINC00494, TSP0AP1-AS1, MCM3AP-AS1, and LINC01588 were downregulated, and OIP5-AS1 was upregulated, consistent with the sequencing data. We further evaluated the diagnostic potential of these five lncRNAs as RA biomarkers and examined their correlations with RA-related clinical indicators.

Studies have indicated that the five dysregulated lncRNAs (LINC00494, TSP0AP1-AS1, MCM3AP-AS1, LINC01588, and OIP5-AS1) may be implicated in RA pathophysiology. LINC00494 was reported to be abnormally expressed in osteonecrosis of the femoral head (15). Moreover, LINC00494 has been found to promote ovarian cancer advancement by regulating FBXO32 via binding and enhancing the activity of the NF-kappaB1 (NF-κB1) (16). NF-κB1 is a core member of the NF-κB family, and its precursor protein p105 is processed to generate an active p50 subunit. p50 usually forms a heterodimer with p65 and is associated with the body’s inflammatory and immune response. Aberrant activation of NF-κB1 causes overexpression of many cytokines and chemokines, leading to the occurrence of RA (17). Recent studies indicated that TSPOAP1-AS1 was downregulated in various cancers and may serve as a groundbreaking treatment focus (18, 19). MCM3AP-AS1 functioned as a competing endogenous RNA, effectively regulating miR-501-3p/STAT3/NF-κB pathway, thereby ameliorating inflammation and mitochondrial function, then alleviating sepsis- caused cardiomyopathy (20). The STAT3 and NF-κB signaling pathways synergistically regulate inflammation, immune response and joint destruction, jointly promoting the progression of RA. In osteoarthritis (OA), MCM3AP-AS1 level was detected to be reduced in OA cartilage tissues and shields chondrocytes from inflammation triggered by interleukin (IL)-1β by regulating the miR-138-5p/SIRT1 signaling pathway (21). Another study evidenced that MCM3AP-AS1 promoted chondrocyte apoptosis of OA patients by modulating the miR-142-p/HMGB1 pathway (22). In pemphigus, an autoimmune skin disease, LINC01588 may epigenetically modulate Th17/Treg equilibrium through the peroxisome proliferator-activated receptor signaling pathway (23). LncRNA OIP5-AS1 is one of the most well-studied lncRNAs. Qin et al. (24) suggested that OIP5-AS1 was found to be upregulated, inhibiting the apoptosis in chondrocytes from OA patients through the suppression of miR-30a-5p activity. In RA-FLS, OIP5-AS1 was upregulated by regulating the miR-410-3p/Wnt7b pathway and activating the Wnt/β-catenin pathway (25, 26). Additionally, OIP5-AS1 may alleviate the progression of RA through the miR-448-PON1-TLR3-NF-κB axis (27). Previously, our group found OIP5-AS1 upregulation in RA plasma exosomes (28). In this study, we found obvious dysregulation in the levels of these five lncRNAs in PBMC from RA patients, with their combination yielding a higher AUC than individual assessments. In addition, LINC00494 showed a negative correlation with RF, MCM3AP-AS1 correlated positively with anti-CCP, and LINC01588 correlated negatively with CRP, suggesting that these lncRNAs may serve as prognostic factors in RA.

A previous study reported that the THUMPD3-AS1 level was significantly reduced in cartilage tissues from OA cases and in chondrocyte cell lines treated with IL-1β (29). Fei et al. (30) verified up-regulation of LINC01094 in peripheral whole blood from osteoporosis patients (OP). Huang et al. (31) indicated that LINC01094 was increased in OA tissues and LPS-induced chondrocytes. In the current research, there has no any differences in the amounts of THUMPD3-AS1 and LINC01094 between people with RA and healthy people. This discrepancy in expression patterns may reflect the distinct pathological mechanisms underlying different types of osteoarthrosis. Although RA, OA, and OP all affect the osteoarticular system, there are significant differences in their pathogenesis: RA is primarily characterized by autoimmune-mediated joint inflammation, OA is predominantly associated with degenerative changes resulting from mechanical stress, and OP is primarily linked to a metabolic imbalance in bone. This finding suggested that these lncRNAs may be disease-specific, enhancing our understanding of the molecular characteristics of various bone and joint diseases.

Notably, certain limitations must be acknowledged in this research. Firstly, the insufficient sample size may restrict the broader applicability of our results. Moreover, the exact roles and mechanisms of these dysregulated lncRNAs in the progression of RA have yet to be fully elucidated, warranting further in-depth research.

In conclusion, the outcomes of our study implied that the expression of lncRNAs (LINC00494, TSP0AP1-AS1, MCM3AP-AS1, LINC01588, and OIP5-AS1) may be changed in the PBMC of RA patients, underscoring their potential utility to serve as biomarkers for RA. These specified lncRNAs present as prospective indicators for RA and exerts important function in deciphering the underlying etiology of RA. It is essential to undertake additional exhaustive exploration to corroborate the specific function of these lncRNAs in RA.

## Data Availability

The datasets generated for this study can be found in the NCBI database GEO GSE291978.

## References

[1] Finckh A Gilbert B Hodkinson B Bae SC Thomas R Deane KD. Global epidemiology of rheumatoid arthritis. Nat Rev Rheumatol. (2022) 18:591–602. doi: 10.1038/s41584-022-00827-y 36068354

[2] Lee YK Ahn GY Lee J Shin JM Lee TH Park DJ. Excess mortality persists in patients with rheumatoid arthritis. Int J Rheum Dis. (2021) 24:364–72. doi: 10.1111/1756-185X.14058 33463890

[3] Lau CS. Burden of rheumatoid arthritis and forecasted prevalence to 2050. Lancet Rheumatol. (2023) 5:e567–e8. doi: 10.1016/s2665-9913(23)00240-0 38251476

[4] Li J Wu GC Zhang TP Yang XK Chen SS Li LJ. Association of long noncoding RNAs expression levels and their gene polymorphisms with systemic lupus erythematosus. Sci Rep. (2017) 7:15119. doi: 10.1038/s41598-017-15156-4 29123179 PMC5680319

[5] Bridges MC Daulagala AC Kourtidis A. LNCcation: lncRNA localization and function. J Cell Biol. (2021) 220:e202009045. doi: 10.1083/jcb.202009045 33464299 PMC7816648

[6] Kumar D Sahoo SS Chauss D Kazemian M Afzali B. Non-coding RNAs in immunoregulation and autoimmunity: Technological advances and critical limitations. J Autoimmun. (2023) 134:102982. doi: 10.1016/j.jaut.2022.102982 36592512 PMC9908861

[7] Ashrafizadeh M Zarrabi A Mostafavi E Aref AR Sethi G Wang L. Non-coding RNA-based regulation of inflammation. Semin Immunol. (2022) 59:101606. doi: 10.1016/j.smim.2022.101606 35691882

[8] Ali SA Peffers MJ Ormseth MJ Jurisica I Kapoor M. The non-coding RNA interactome in joint health and disease. Nat Rev Rheumatol. (2021) 17:692–705. doi: 10.1038/s41584-021-00687-y 34588660

[9] Shui X Chen S Lin J Kong J Zhou C Wu J. Knockdown of lncRNA NEAT1 inhibits Th17/CD4(+) T cell differentiation through reducing the STAT3 protein level. J Cell Physiol. (2019) 234:22477–84. doi: 10.1002/jcp.28811 31119756

[10] Rao Y Fang Y Tan W Liu D Pang Y Wu X. Delivery of long non-coding RNA NEAT1 by peripheral blood monouclear cells-derived exosomes promotes the occurrence of rheumatoid arthritis via the microRNA-23a/MDM2/SIRT6 axis. Front Cell Dev Biol. (2020) 8:551681. doi: 10.3389/fcell.2020.551681 33042992 PMC7517357

[11] Mu N Gu JT Huang TL Liu NN Chen H Bu X. Blockade of discoidin domain receptor 2 as a strategy for reducing inflammation and joint destruction in rheumatoid arthritis via altered interleukin-15 and dkk-1 signaling in fibroblast-like synoviocytes. Arthritis Rheumatol. (2020) 72:943–56. doi: 10.1002/art.41205 32362074

[12] Wang J Zhao Q. LncRNA LINC-PINT increases SOCS1 expression by sponging miR-155-5p to inhibit the activation of ERK signaling pathway in rheumatoid arthritis synovial fibroblasts induced by TNF-α. Int Immunopharmacol. (2020) 84:106497. doi: 10.1016/j.intimp.2020.106497 32289665

[13] Aletaha D Neogi T Silman AJ Funovits J Felson DT Bingham CO 3rd. 2010 rheumatoid arthritis classification criteria: an American College of Rheumatology/European League Against Rheumatism collaborative initiative. Ann Rheum Dis. (2010) 69:1580–8. doi: 10.1136/ard.2010.138461 20699241

[14] Sigdel KR Cheng A Wang Y Duan L Zhang Y. The emerging functions of long noncoding RNA in immune cells: autoimmune diseases. J Immunol Res. (2015) 2015:848790. doi: 10.1155/2015/848790 26090502 PMC4451983

[15] Han N Li Z. Non-coding RNA identification in osteonecrosis of the femoral head using competitive endogenous RNA network analysis. Orthop Surg. (2021) 13:1067–76. doi: 10.1111/os.12834 PMC8126913 33749138

[16] Shu Y Zhang H Li J Shan Y. LINC00494 promotes ovarian cancer development and progression by modulating NFκB1 and FBXO32. Front Oncol. (2021) 10:541410. doi: 10.3389/fonc.2020.541410 33585183 PMC7877250

[17] Ding Q Hu W Wang R Yang Q Zhu M Li M. Signaling pathways in rheumatoid arthritis: implications for targeted therapy. Signal Transduct Target Ther. (2023) 8:68. doi: 10.1038/s41392-023-01331-9 36797236 PMC9935929

[18] Ma C Li F Gu Z Yang Y Qi Y. A novel defined risk signature of cuproptosis-related long non-coding RNA for predicting prognosis, immune infiltration, and immunotherapy response in lung adenocarcinoma. Front Pharmacol. (2023) 14:1146840. doi: 10.3389/fphar.2023.1146840 37670938 PMC10475834

[19] Tang X Zhang M Sun L Xu F Peng X Zhang Y. The biological function delineated across pan-cancer levels through lncRNA-based prognostic risk assessment factors for pancreatic cancer. Front Cell Dev Biol. (2021) 9:694652. doi: 10.3389/fcell.2021.694652 34195204 PMC8236889

[20] Nie X Deng W Zhou H Wang Z. Long noncoding RNA MCM3AP-AS1 attenuates sepsis-induced cardiomyopathy by improving inflammation, oxidative stress, and mitochondrial function through mediating the miR-501-3p/CADM1/STAT3 axis. Int Immunopharmacol. (2024) 128:111500. doi: 10.1016/j.intimp.2024.111500 38237222

[21] Shi J Cao F Chang Y Xin C Jiang X Xu J. Long non-coding RNA MCM3AP-AS1 protects chondrocytes ATDC5 and CHON-001 from IL-1β-induced inflammation via regulating miR-138-5p/SIRT1. Bioengineered. (2021) 12:1445–56. doi: 10.1080/21655979.2021.1905247 PMC8806229 33942704

[22] Gao Y Zhao H Li Y. LncRNA MCM3AP-AS1 regulates miR-142-3p/HMGB1 to promote LPS-induced chondrocyte apoptosis. BMC Musculoskelet Disord. (2019) 20:605. doi: 10.1186/s12891-019-2967-4 31836002 PMC6911297

[23] Huang ZX Qu P Wang KK Zheng J Pan M Zhu HQ. Transcriptomic profiling of pemphigus lesion infiltrating mononuclear cells reveals a distinct local immune microenvironment and novel lncRNA regulators. J Transl Med. (2022) 20:182. doi: 10.1186/s12967-022-03387-7 35449056 PMC9027862

[24] Qin GH Yang WC Yao JN Zhao Y Wu XJ. LncRNA OIP5-AS1 affects the biological behaviors of chondrocytes of patients with osteoarthritis by regulating micro-30a-5p. Eur Rev Med Pharmacol Sci. (2021) 25:1215–24. doi: 10.26355/eurrev_202102_24825 33629291

[25] Pan L Sun Y Jiang H Chen Y Jiang Y Han Y. Total saponins of radix clematis regulate fibroblast-like synoviocyte proliferation in rheumatoid arthritis via the lncRNA OIP5-AS1/miR-410-3p/wnt7b signaling pathway. Evid Based Complement Alternat Med. (2022) 2022:8393949. doi: 10.1155/2022/8393949 35668775 PMC9166986

[26] Sun Y Jiang H Pan L Han Y Chen Y Jiang Y. LncRNA OIP5-AS1/miR-410-3p/Wnt7b axis promotes the proliferation of rheumatoid arthritis fibroblast-like synoviocytes via regulating the Wnt/β-catenin pathway. Autoimmunity. (2023) 56:2189136. doi: 10.1080/08916934.2023.2189136 36942896

[27] Qing P Liu Y. Inhibitory role of long non-coding RNA OIP5-AS1 in rheumatoid arthritis progression through the microRNA-448-paraoxonase 1-toll-like receptor 3-nuclear factor κB axis. Exp Physiol. (2020) 105:1708–19. doi: 10.1113/ep088608 32770578

[28] Shuai ZQ Wang ZX Ren JL Yang XK Xu B. Differential expressions and potential clinical values of lncRNAs in the plasma exosomes of rheumatoid arthritis. Int Immunopharmacol. (2024) 128:111511. doi: 10.1016/j.intimp.2024.111511 38194746

[29] Wang Y Li T Yang Q Feng B Xiang Y Lv Z. LncRNA THUMPD3-AS1 enhances the proliferation and inflammatory response of chondrocytes in osteoarthritis. Int Immunopharmacol. (2021) 100:108138. doi: 10.1016/j.intimp.2021.108138 34509934

[30] Fei Q Bai X Lin J Meng H Yang Y Guo A. Identification of aberrantly expressed long non-coding RNAs in postmenopausal osteoporosis. Int J Mol Med. (2018) 41:3537–50. doi: 10.3892/ijmm.2018.3575 PMC5881766 29568943

[31] Huang F Su Z Yang J Zhao X Xu Y. Knocking-down long non-coding RNA LINC01094 prohibits chondrocyte apoptosis via regulating microRNA-577/metal-regulatory transcription factor 1 axis. J Orthop Surg (Hong Kong). (2024) 32:10225536241254588. doi: 10.1177/10225536241254588 38758016

